# Prevalence and prognosis of non-dilated left ventricular cardiomyopathy in patients referred for cardiac magnetic resonance

**DOI:** 10.1007/s00330-025-12072-4

**Published:** 2025-10-28

**Authors:** Rungroj Krittayaphong, Thammarak Songsangjinda, Yodying Kaolawanich, Kanchalaporn Jirataiporn, Ahthit Yindeengam

**Affiliations:** 1 Division of Cardiology, Department of Medicine, Faculty of Medicine Siriraj Hospital, Mahidol University, Bangkok, Thailand; 2 Cardiology Unit, Division of Internal Medicine, Faculty of Medicine, Prince of Songkla University, Songkhla, Thailand; 3 Her Majesty’s Cardiac Center, Faculty of Medicine Siriraj Hospital, Mahidol University, Bangkok, Thailand

**Keywords:** Magnetic resonance imaging, Dilated cardiomyopathy, Heart failure

## Abstract

**Background:**

The European Society of Cardiology recently identified non-dilated left ventricular cardiomyopathy (NDLVC) as a distinct clinical entity. This study aimed to determine the prevalence and prognosis of NDLVC in a specific cohort.

**Materials and methods:**

This retrospective cohort analysis included patients referred for routine cardiac magnetic resonance (CMR) and who underwent stress myocardial perfusion or viability assessment from September 2017 to July 2020. Patients with coronary artery disease (CAD) from history or CMR imaging were excluded. Participants were categorized into dilated cardiomyopathy (DCM), NDLVC, and control groups. NDLVC was defined as the presence of non-ischemic LGE or isolated global left ventricular hypokinesia without left ventricular dilatation. The primary endpoints were all-cause mortality or heart failure events.

**Results:**

Among 4377 CMR patients, 2278 patients remain after the exclusion. Mean age was 66.3 ± 13.2 years, and 60.4% were females. Upon categorization, there were 1996 controls (87.6%), 172 DCM patients (7.6%), and 110 NDLVC patients (4.8%). Among the NDLVC patients, 64 patients (58.2%) had non-ischemic LGE, which included mid-wall, subepicardial, patchy and right ventricular insertion types. Over a median follow-up of 37.5 months, DCM patients exhibited higher rates of composite outcomes than NDLVC and control patients. Likewise, NDLVC patients also had significantly greater rates of composite outcomes and heart failure events than the controls.

**Conclusion:**

NDLVC had a prevalence of 2.5% among routine stress or viability CMR cases and had a significantly higher rate of clinical outcomes than the controls but lower than DCM patients. These findings underscore the distinct clinical significance of NDLVC.

**Key Points:**

***Question*** *What is the prevalence and clinical prognosis of non-dilated left ventricular cardiomyopathy (NDLVC) in patients undergoing routine stress or viability cardiac MRI?*

***Findings*** *NDLVC was present in 2.5% of patients undergoing routine stress or viability cardiac MRI and was associated with worse clinical outcomes than controls, but more favorable than dilated cardiomyopathy.*

***Clinical relevance*** *Identifying NDLVC on routine cardiac magnetic resonance (CMR) provides prognostic value, helping to recognize patients at increased risk of heart failure events despite preserved left ventricular size.*

**Graphical Abstract:**

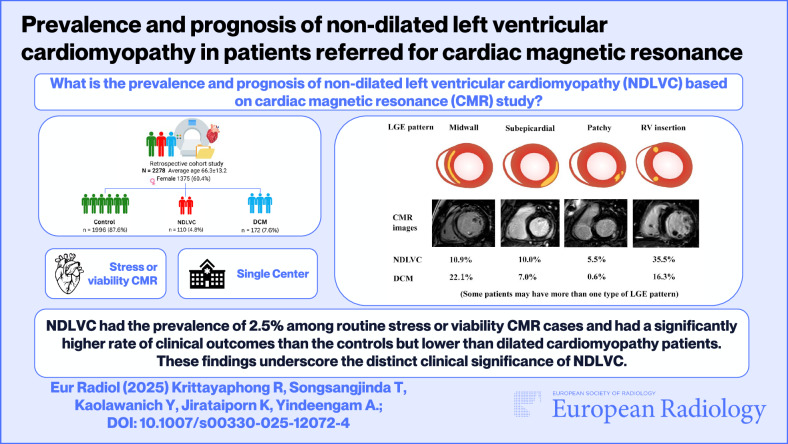

## Introduction

The classification of non-ischemic cardiomyopathy is increasingly challenging, with perspectives evolving and criteria being reconsidered over time. In 2006, the American Heart Association introduced a classification scheme for cardiomyopathies, segregating them into genetic (e.g., hypertrophic cardiomyopathy [HCM]), mixed (e.g., dilated cardiomyopathy [DCM]) and acquired (e.g., peripartum cardiomyopathy) types [1]. In 2014, the MOGES classification emerged, aiding clinicians in distinguishing cardiomyopathy types by focusing on morpho-functional phenotypes, organ/system involvement, genetic inheritance, etiological factors and disease stages [2].

The European Society of Cardiology (ESC) recently recommended contrast-enhanced cardiac magnetic resonance (CMR) as the initial assessment tool for suspected cardiomyopathies. These guidelines recommend contrast-enhanced CMR as the initial assessment tool for suspected cardiomyopathies (Class I recommendation) and for ongoing evaluation to monitor disease progression, risk stratification and management guidance (Class IIa recommendation). The guidelines also urge physicians to adopt a cardiomyopathy-focused approach when evaluating patients. Cardiomyopathies are classified based on distinct morphological patterns and functional assessments. Additionally, CMR facilitates the assessment of myocardial mechanics using feature-tracking techniques. The prognostic value of LV global longitudinal strain (LV-GLS) and left atrial strain (LAS), ascertained through CMR, has been established in patients with cardiovascular disease [3, 4]. The guideline delineates specific cardiomyopathy phenotypes: HCM, DCM, non-dilated left ventricular cardiomyopathy (NDLVC), arrhythmogenic right ventricular cardiomyopathy (ARVC) and restrictive cardiomyopathy [5].

The ESC guidelines have refined the cardiomyopathy nomenclature, adopting NDLVC over the term hypokinetic non-dilated cardiomyopathy (HNDC), which was featured in earlier ESC directives for defining DCM [6]. In the 2022 guidelines, HNDC was characterized by left ventricular (LV) hypokinesia without dilatation [7]. HNDC was proposed as part of the spectrum of DCM. However, the latest ESC guidelines endorse CMR as the primary diagnostic modality for initial assessment. The guidelines stipulate that late gadolinium enhancement (LGE) identification should be integral to cardiomyopathy phenotyping [5]. Consequently, NDLVC is currently defined as the presence of non-ischemic LGE (with or without global or regional wall motion abnormalities) or as isolated global LV hypokinesia without LGE [5]. Despite being recognized as a distinct entity different from DCM at present, there is limited evidence confirming the difference in prognosis within this disease spectrum.

This study primarily aimed to determine the prevalence and prognosis of NDLVC compared to DCM and control groups in patients undergoing CMR for routine myocardial perfusion or viability assessment. The secondary objective was to determine the differences in LV-GLS, LAS, and extracellular volume fraction (ECV) derived via CMR feature-tracking and T1 mapping, among patients with NDLVC or DCM and controls. Since NDLVC is a relatively new clinical and imaging condition, this study’s findings will offer valuable insights to help answer important clinical questions and highlight the significance of this specific disorder.

## Materials and methods

### Study population

This study represents a retrospective cohort analysis. We evaluated patients older than 18 years who were referred for CMR imaging to assess myocardial stress perfusion or viability from July 2017 to July 2020. The exclusion criteria were a history of myocardial infarction or coronary revascularization; a positive ischemic LGE identified by CMR (manifesting as a subendocardial or transmural LGE); myocardial perfusion defects; a diagnosis compatible with HCM or ARVC; and isolated LV dilatation without a reduced LV ejection fraction (LVEF) or LGE. For patients who underwent multiple CMR examinations, the initial CMR scan was selected. Ethical approval for this study was obtained from the Institutional Review Board of Siriraj Hospital. Informed consent was not required due to the retrospective nature of the study. Then, patients were assigned to DCM, NDLVC, and controls. DCM patients were those with LV dilatation and low LVEF. NDLVC patients were those with low LVEF without LV dilatation or those with non-ischemic type LGE without LV dilatation. Patients with LV dilatation without low LVEF were excluded. The control group was patients with normal LVEF without LV dilatation and without LGE.

Baseline characteristics were recorded. Definitions of cardiovascular risk variables were as follows: Current smoker, (smoke at least 1 cigarette per day at least 1 month, or stopped less than 3 months prior); Dyslipidemia included at least one of the following; total cholesterol (TC) > 200 mg/dL (5.18 mmol/L), LDL-C > 130 mg/dL (3.37 mmol/L), HDL-C < 40 mg/dL (1.04 mmol/L) or TG > 150 mg/dL; Diabetes mellitus, fasting plasma glucose at least 126 mg/dL at least twice, HbA1C at least 6.5%, random plasma glucose at least 200 mg/dL, or being treated with antihyperglycemic medication; Hypertension: systolic blood pressure 140 mmHg, or diastolic blood pressure 90 mmHg on at least 2 visits, or being treated with antihypertensive medications.

### Cardiac magnetic resonance protocol

CMR imaging was performed using a 3-Tesla Ingenia scanner (Philips Medical Systems) with a 32-channel coil and electrocardiogram gating. The CMR protocol included black blood axial imaging, adenosine-induced stress perfusion scans, LGE studies and steady-state free precession cine sequences. The cine sequences were captured in 25 phases of the cardiac cycle, with an 8 mm slice thickness without inter-slice gaps, using breath-hold techniques and retrospective electrocardiogram gating. The scanning parameters were as follows: echo time, 1.4 ms; repetition time, 2.9 ms; flip angle, 45°; field of view, 270 × 320 mm²; and acquired voxel size, 1.5 × 1.4 × 8 mm³. The temporal resolution achieved was approximately 34 ± 6 ms. A first-pass perfusion study was conducted during stress CMR using an injection of 0.05 mmol/kg of gadolinium contrast agent. An additional 0.1 mmol/kg of gadolinium was injected immediately after the acquisition of the perfusion images.

Native and post-contrast T1 mapping was performed using the modified look-locker inversion sequence with a 5-(3)-3 scheme [8, 9]. The sequence was acquired during breath-hold in mid-diastole with a single mid-ventricular short-axis slice. The following are the scanning parameters: echo time, 1.8 ms; repetition time, 2.2 ms; 8 inversion times; field of view, 300 × 300 mm; matrix size, 152 × 150; and slice thickness, 10 mm; flip angle, 20°; SENSE factor, 2.

### Analysis of LV function and LGE

CMR data analysis was interpreted by experienced CMR interpreters, each with more than 5 years of experience. The interpreters were blinded to patient identifiers, CMR timings and clinical information. Epicardial and endocardial borders were manually outlined on each short-axis image to define myocardial contours. The LVEF was determined using end-systolic and end-diastolic volumes derived from these short-axis slices. We used the suggested normal reference values stratified by age and sex [10] to determine left ventricular dilatation. The LGE images were assessed visually. The presence of LGE was recorded, and the patterns were categorized as ischemic LGE for subendocardial or transmural patterns and as non-ischemic LGE for mid-wall, subepicardial, patchy or right ventricular insertion point patterns.

Eligible patients were then categorized into 3 groups according to CMR findings as follows: DCM group, defined as the presence of LV dilatation with reduced global LV systolic function; NDLVC group, defined as the presence of non-ischemic LGE or LV systolic dysfunction in the absence of LV dilatation [5]; and control group including the rest who had non-dilated LV with normal LV systolic function in the absence of LGE.

### LV-GLS and LAS assessments

Cine CMR images were processed using 4-chamber, 3-chamber, and 2-chamber views, essentially for LV-GLS and LAS quantification. LV-GLS was then determined using feature-tracking analysis with CVI42 version 5.13 software (Circle Cardiovascular Imaging Inc.), as described in the literature [11]. The feature-tracking software initially performed automated tracking of the myocardial boundaries, which were manually refined to ensure accurate delineation of the region of interest. This process was crucial for encompassing the entire myocardial thickness in the measurements.

The LV-GLS measurements were taken at the end-diastolic phase and are therefore reported as negative values. A greater negative magnitude in these values, that is, more negative numerically, indicated better myocardial function. To ensure objectivity, CMR imaging and LV-GLS measurements were performed without access to the patients’ clinical data. This approach guaranteed that the measurements were unbiased and not influenced by any prior clinical information about the patients.

Patients with atrial fibrillation were excluded from LAS analysis to avoid confounding effects. The endocardial and epicardial borders of the left atrium were manually traced on 2- and 4-chamber long-axis images, adding an extrapolation line at the orifices of the pulmonary veins and the left atrial appendage. Ventricular end-diastole was set as the baseline for strain measurements, in line with EACVI/ASE/Industry Task Force recommendations [12]. Manual adjustments were made to ensure accurate tracking. Peak LAS values were derived from the difference between the peak strain and the baseline on the longitudinal strain‒time curve.

### Analysis of extracellular volume fraction

CVI42 software (version 5.12, Circle Cardiovascular Imaging) was used for the analysis of native and contrast-enhanced T1 mapping. A region of interest was carefully drawn for the entire interventricular septum of the mid-cavity short-axis slice, with the avoidance of imaging artifacts. According to the guidelines of the Society for Cardiovascular Magnetic Resonance [9], the region of interest for T1 mapping can be the septal segments or a complete, single, short-axis slice. The ECV was calculated using the following formula [13]:

$${{\rm{ECV}}}\left( \% \right)=	\, (1-{{\rm{Hematocrit}}}) \\ 	 \times \left(\frac{\frac{1}{{{\rm{Post\; contrast}}}\,{{{\rm{T}}}1}_{{{\rm{Myocardium}}}}}-\frac{1}{{{\rm{Native}}}\,{{{\rm{T}}}1}_{{{\rm{Myocardium}}}}}}{\frac{1}{{{\rm{Post\; contrast}}}\,{{{\rm{T}}}1}_{{{\rm{Blood}}}}}-\frac{1}{{{\rm{Native}}}\,{{{\rm{T}}}1}_{{{\rm{Blood}}}}}}\right)\times 100$$

ECV = extracellular volume fraction (proportion of the myocardial tissue volume that is occupied by the extracellular (interstitial) space), Hematocrit = actual hematocrit, post-contrast T1 = Post-contrast T1 time of the myocardium, Native T1Myocardium = Native (pre-contrast) T1 time of the myocardium (in milliseconds), Post-contrast T1Blood = Post-contrast T1 time of the blood pool, Native T1Blood = Native (pre-contrast) T1 time of the blood pool.

### Outcomes

To minimize bias, outcomes were determined through electronic medical record analyses conducted by evaluators who were blinded to the clinical data and CMR results. This was supplemented by structured telephone interviews. The primary composite outcome of the study was defined as all-cause mortality or a heart failure event, with each outcome analyzed separately. A heart failure event was stringently defined as requiring a hospital admission of more than 24 h. This criterion was applied to both patients who presented with new-onset heart failure and those who experienced worsening heart failure symptoms. The diagnosis was supported by clinical signs indicative of heart failure and corroborative evidence from laboratory assessments (such as elevated natriuretic peptides) or imaging (such as chest radiographs showing increased pulmonary congestion). Receiving treatment for heart failure during hospital admission was also a necessary criterion for the diagnosis.

### Statistical analysis

Descriptive statistics were used to summarize the baseline characteristics of the cohort. Continuous variables are presented as means with standard deviations, and one-way ANOVA (with Bonferroni post hoc analysis) was used for comparative analysis. Categorical variables are expressed as frequencies and percentages, and the chi-squared test was used for comparisons.

The incidence rates of clinical outcomes were calculated per 100 person-years, with group comparisons made using Poisson distribution methods. Kaplan–Meier survival analysis and log-rank test comparisons were used to evaluate the differences in outcomes between the study groups.

Univariable and multivariable Cox proportional hazards regression models were used to determine whether NDLVC and DCM independently predicted the study outcomes. The variables adjusted for potential confounding factors were demographic data and cardiovascular risk factors, i.e., age, sex, smoking status; being diagnosed with dyslipidemia, type 2 diabetes mellitus, and hypertension.

A sensitivity analysis was conducted using a restricted control group (restricted control group sensitivity analysis), excluding individuals with any history of heart failure, atrial fibrillation, or stroke, to assess the robustness of the primary findings. Data analyses were conducted using IBM SPSS Statistics, version 28 (IBM Corp); MedCalc Statistical Software, version 20 (MedCalc Software Ltd); and R statistical software, version 4.2.3 (www.r-project.org).

## Results

### Study population

**Fig. 1:**
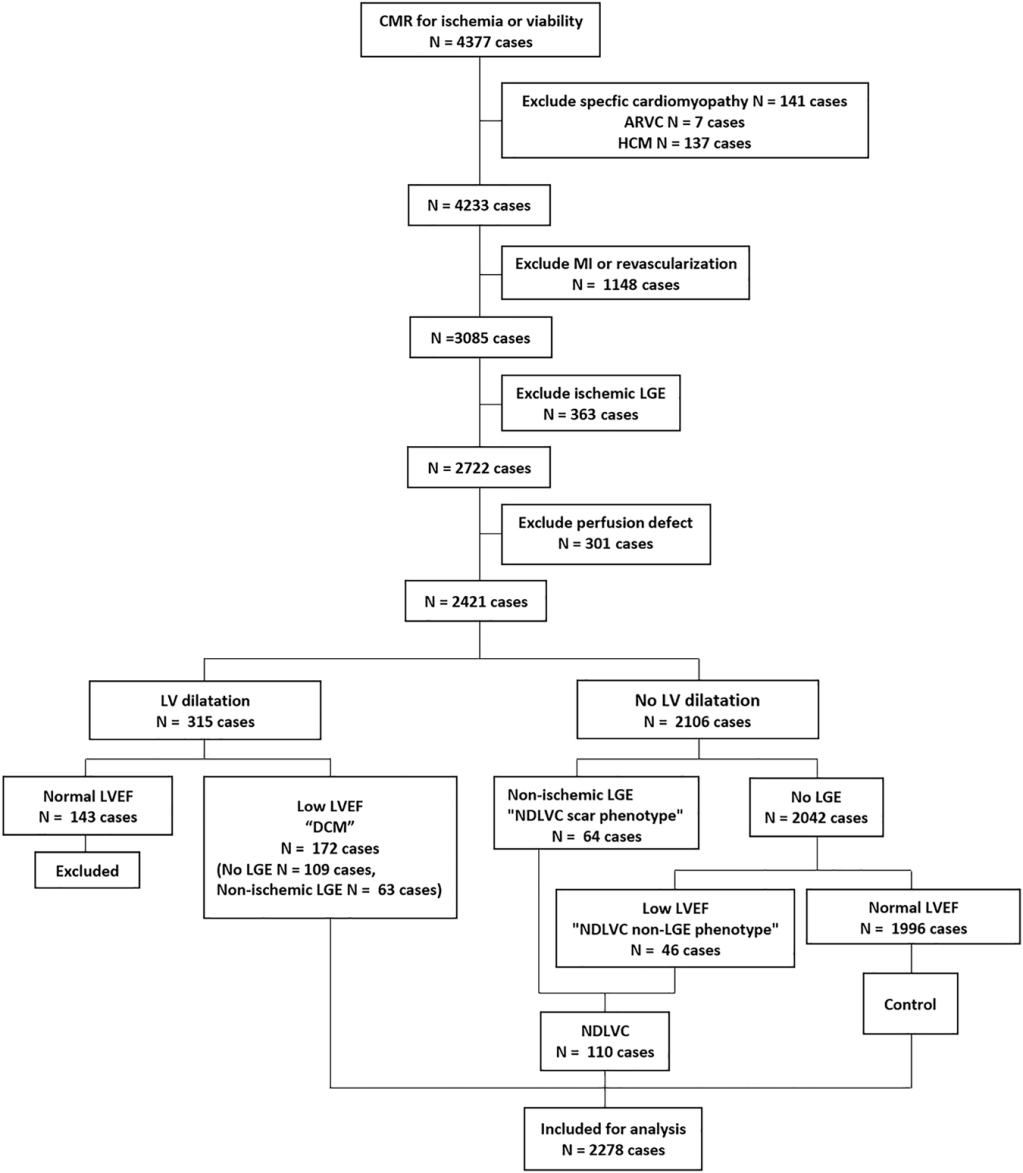
Study population flowchart detailing participant selection and exclusion. ARVC, arrhythmogenic right ventricular cardiomyopathy; CAD, coronary artery disease; CMR, cardiac magnetic resonance; DCM, dilated cardiomyopathy; HCM, hypertrophic cardiomyopathy; LV, left ventricular; LVEF, left ventricular ejection fraction; MI, myocardial infarction; NDLVC, non-dilated left ventricular cardiomyopathy

After applying the exclusion criteria, the study included 2278 eligible patients from the initial pool of 4377 who underwent CMR for stress myocardial perfusion or myocardial viability assessment (Fig. 1). The mean age of the individuals in the cohort was 66.3 ± 13.2 years, and 1375 (60.4%) were females. Classification yielded 7.6% (172) with DCM, 4.8% (110) with NDLVC and 87.6% (1996) as controls. The prevalence of NDLVC among the initial 4377 patients who underwent CMR was 2.5% (110 patients) and accounted for 39% of the combined NDLVC and DCM cases (110 out of 282 patients). The followings are indications for CMR (each patient may have more than 1): dyspnea (*n* = 813); chest pain (*n* = 392); history of heart failure (*n* = 210); abnormal ECG (such as Q-wave, ST-T changes or left ventricular hypertrophy) (*n* = 493); abnormal echocardiogram (such as reduced left ventricular systolic function, abnormal wall motion) (*n* = 184) positive, equivocal or inconclusive exercise test (*n* = 234); and preoperative assessment (*n* = 144).

The baseline characteristics, detailed in Table 1, revealed that the DCM patients were younger and had fewer cardiovascular risk factors than did the other two groups. However, the DCM group more frequently had a history of heart failure. In contrast, a history of atrial fibrillation was more common in NDLVC patients.

**Table 1:** Baseline demographic and clinical characteristics of the study participants

Characteristics	All (*N* = 2278)	Control (*n* = 1996)	NDLVC (*n* = 110)	DCM (*n* = 172)	*p*-value*
Age (years)	66.3 ± 13.2	67.1 ± 12.4	64.3 ± 15.1	58.4 ± 17.4	< 0.001^a,c^
Female sex	1375 (60.4%)	1241 (62.2%)	48 (43.6%)	86 (50%)	0.001^a,b^
BMI (kg/m^2^)	25.9 ± 4.9	26.1 ± 4.8	25.4 ± 4.9	24.3 ± 5.7	< 0.001^a^
Current smoker	49 (2.2%)	39 (2.0%)	4 (3.6%)	6 (3.5%)	0.225
Dyslipidemia	1387 (60.9%)	1284 (64.3%)	53 (48.2%)	50 (29.1%)	< 0.001^a,b,c^
Diabetes mellitus	759 (33.3%)	681 (34.1%)	43 (39.1%)	35 (20.3%)	< 0.001^a,c^
Hypertension	1561 (68.5%)	1404 (70.3%)	66 (60%)	91 (52.9%)	< 0.001^a^
Family history of CAD	15 (0.7%)	14 (0.7%)	0.0 (0.0%)	1 (0.6%)	0.670
History of heart failure	210 (9.2%)	124 (6.2%)	23 (20.9%)	63 (36.6%)	< 0.001^a,b,c^
History of atrial fibrillation	230 (10.1%)	162 (8.1%)	34 (30.9%)	34 (19.8%)	< 0.001^a,b^
History of stroke	140 (6.1%)	124 (6.2%)	5 (4.5%)	11 (6.4%)	0.770
Ischemic stroke	135 (5.9%)	119 (6.0%)	5 (4.5%)	11 (6.4%)	
Unknown	5 (0.2%)	5 (0.3%)	0 (0.0%)	0 (0.0%)	
Chronic kidney disease	612 (26.9%)	524 (26.3%)	34 (30.9%)	54 (31.4%)	0.213
Symptoms					
Dyspnea on exertion	813 (35.7%)	758 (38%)	23 (20.9%)	32 (18.6%)	< 0.001^a,b^
Chest pain	392 (17.2%)	372 (18.6%)	13 (11.8%)	7 (4.1%)	< 0.001^a,c^

*CAD* coronary artery disease, *DCM* dilated cardiomyopathy, *NDLVC* non-dilated left ventricular cardiomyopathy

* *p*-value of the comparisons of the 3 groups was from ANOVA for continuous data and chi-square for categorical data.

^a^ Significant difference between DCM and control, post hoc analysis

^b^ Significant difference between NDLVC and control, post hoc analysis

^c^ Significant difference between DCM and NDLVC, post hoc analysis

### CMR findings

Among the 110 patients with NDLVC, 50% (55) had non-ischemic LGE with a normal LVEF, 8.2% (9) had non-ischemic LGE with a reduced LVEF, and 41.8% (46) had a reduced LVEF without any LGE. Overall, 58.2% (64) of the NDLVC patients exhibited non-ischemic LGE. Within this subset, the distribution of LGE types was as follows: mid-wall in 18.8% (12) of the patients, subepicardial in 17.2% (11), patchy in 9.4% (6), and right ventricular insertion site in 60.9% (39). Notably, 6.3% (4) had more than one type of non-ischemic LGEs.

**Fig. 2:**
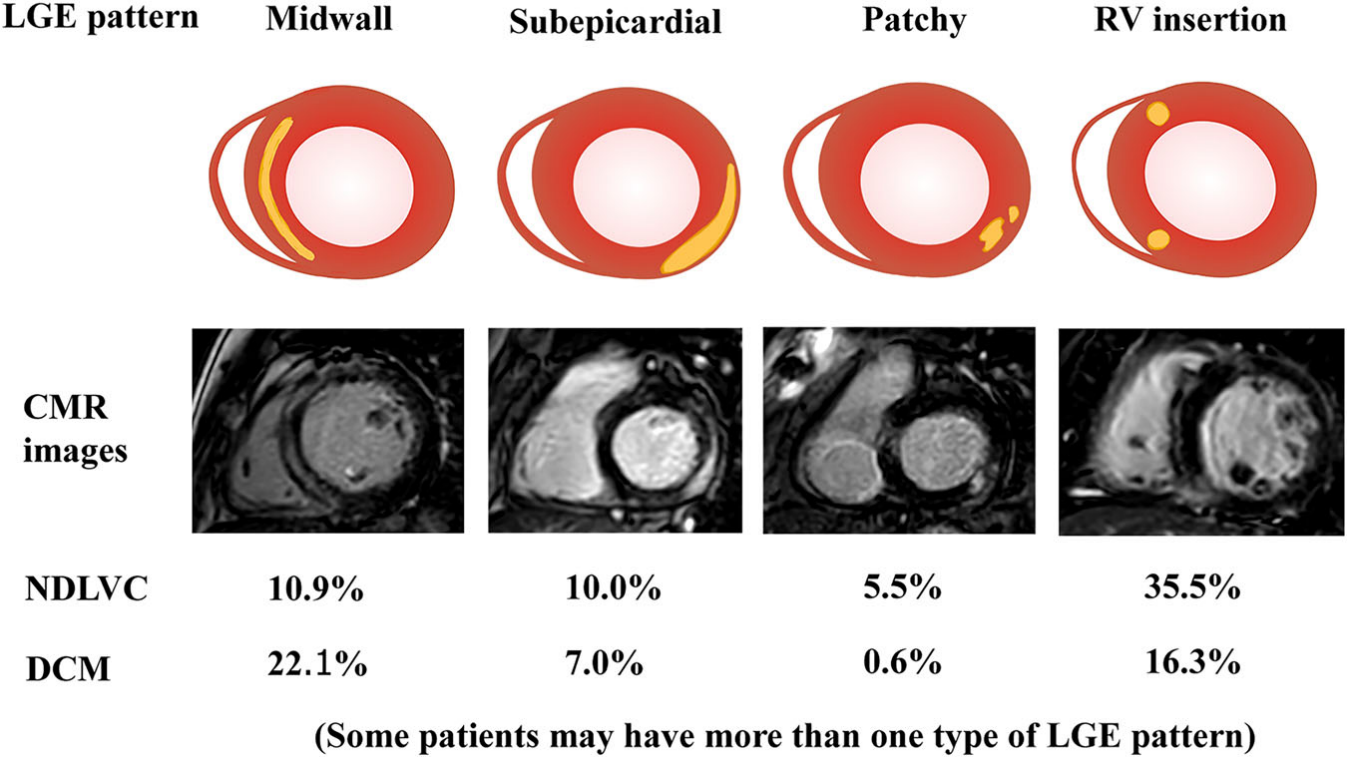
Distribution of non-ischemic myocardial LGE patterns compared between non-dilated left ventricular cardiomyopathy and dilated cardiomyopathy. CMR, cardiac magnetic resonance; DCM, dilated cardiomyopathy; NDLVC, non-dilated left ventricular cardiomyopathy

Of the 172 patients with DCM, 36.6% (63) had non-ischemic LGE. Within this subgroup, the LGE distribution was as follows: mid-wall in 60.3% (38) of the patients, subepicardial in 19.0% (12), patchy in 1.6% (1), and right ventricular insertion site in 44.4% (28). Notably, 25.4% (16) of the patients in the DCM group with non-ischemic LGE had more than one type of LGE, with the most common combination being mid-wall and right ventricular insertion site LGE. The distributions of LGE patterns in the NDLVC and DCM groups are illustrated in Table 2 and Fig. 2. Patients with NDLVC are more likely to have subepicardial, patchy, or RV insertion LGE compared to DCM.

**Table 2:** Distribution of non-coronary artery disease myocardial LGE patterns in non-dilated left ventricular cardiomyopathy and dilated cardiomyopathy patients

	All non-ischemic LGE (*N* = 127)	NDVLC with non-ischemic LGE (*n* = 64)	DCM with non-ischemic LGE (*n* = 63)	*p*-value
Mid-wall LGE alone	34 (26.8%)	9 (14.1%)	25 (39.7%)	0.110
Subepicardial LGE alone	13 (10.2%)	9 (14.1%)	4 (6.3%)	0.022
Patchy LGE alone	5 (3.9%)	5 (7.8%)	0 (0%)	0.005
RV insertion LGE alone	55 (43.3%)	37 (57.8%)	18 (28.6%)	< 0.001
Mid-wall + subepicardial LGE	7 (5.5%)	2 (3.1%)	5 (7.9%)	0.567
Mid-wall + RV insertion LGE	9 (7.1%)	1 (1.6%)	8 (12.7%)	0.081
Subepicardial + patchy LGE	1 (0.8%)	0 (0%)	1 (1.6%)	0.423
Subepicardial + RV insertion LGE	2 (1.6%)	0 (0%)	2 (3.2%)	0.256
Patchy + RV insertion LGE	1 (0.8%)	1 (1.6%)	0 (0%)	0.210

*DCM* dilated cardiomyopathy, *NDLVC* non-dilated left ventricular cardiomyopathy, *non-CAD* non-coronary artery disease, *RV* right ventricular

Control group had no LGE, and had a significantly higher LVEF (73.0 ± 8.3%) compared to NDLVC (56.2 ± 16.7%) and DCM (33.5 ± 11.5%) (*p* < 0.001 by ANOVA test). NDLVC group had a higher LVEF compared to DCM (*p* < 0.001 by post hoc analysis of ANOVA test). Control group had a significantly lower left ventricular end-diastolic volume LVEDV (66.8 ± 11.5 mL) compared to NDLVC (74.2 ± 15.5 mL) and DCM (138.4 ± 38.9 mL) (*p* < 0.001 by ANOVA test). NDLVC group had a lower LVEDV compared to DCM (*p* < 0.001 by post hoc analysis of ANOVA test).

### Outcomes

**Fig. 3:**
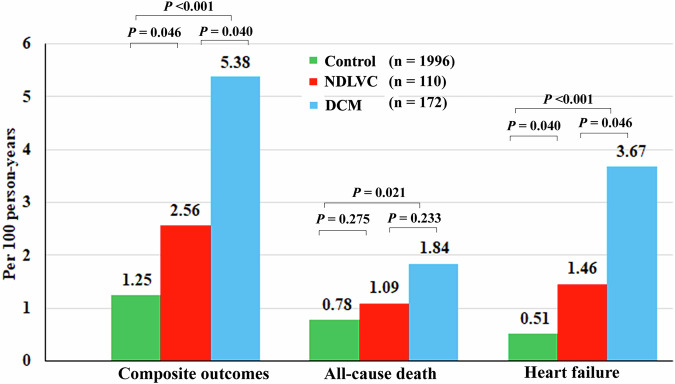
Incidence rates of clinical outcomes in the control, non-dilated left ventricular cardiomyopathy and dilated cardiomyopathy groups

**Fig. 4:**
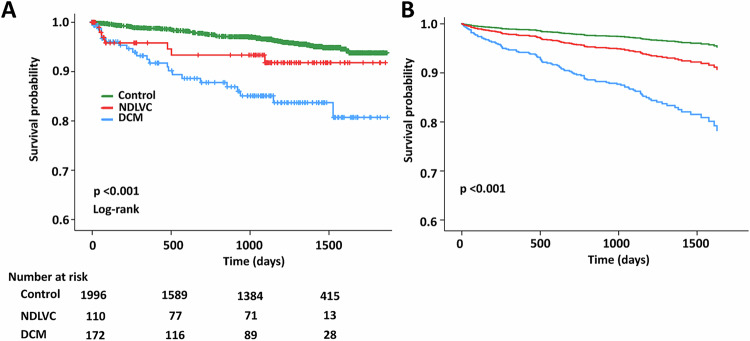
**A** Kaplan–Meier curves depicting event-free survival in the control, non-dilated left ventricular cardiomyopathy and dilated cardiomyopathy groups. **B** Survival graphs of the 3 groups fitted with possible confounding factors, including age, sex, hypertension, diabetes, dyslipidemia, and smoking status. DCM, dilated cardiomyopathy; NDLVC, non-dilated left ventricular cardiomyopathy

Median follow-up period was 37.5 months (interquartile range 23.6–47.0). Out of 2278 patients, 100 (4.4%) patients experienced a composite outcome of all-cause mortality or heart failure;56 (2.5%) died, and 48 (2.1%) experienced heart failure. The incidence rates per 100 person-years of composite outcomes were 5.38 (95% CI 3.37–8.14) in the DCM group, 2.56 (95% CI 1.03–5.28) in the NDLVC group and 1.25 (95% CI 0.97–1.57) in the control group. Table 3 and Fig. 3 detail the incidence rates and comparisons among the three groups. Patients with DCM had higher incidence rates of composite outcomes and heart failure events than the other two groups (*p* < 0.001 compared to control group and *p* = 0.040 compared to NDLVC). Similarly, the NDLVC group demonstrated a significantly greater incidence of composite outcomes and heart failure events than the control group (*p* = 0.046). However, only patients in the DCM group experienced all-cause death in comparison to the control group, while no significant difference in mortality was observed between the NDLVC group and the control group. Figure 4A shows the Kaplan–Meier curve illustrating that the event-free survival for the NDLVC group was intermediate between that of the DCM group and the control group, with the long-rank *p*-value < 0.001. Figure 4B demonstrates survival graphs of the 3 groups fitted with possible confounding factors, including age, sex, hypertension, diabetes, dyslipidemia, and smoking status, confirming the results of the Kaplan–Meier analysis with the *p*-value < 0.001 from the Cox model.

**Table 3:** Incidence rates of composite outcomes, all-cause mortality, and heart failure events in the control, dilated cardiomyopathy and non-dilated left ventricular cardiomyopathy groups

	Number of patients	Number of events	100 person-years	Rate per 100 person-years
Composite outcomes	2278	100	63.78	1.57 (1.28–1.91)
Control	1996	71	56.96	1.25 (0.97–1.57)
NDLVC	110	7	2.73	2.56 (1.03–5.28)
DCM	172	22	4.09	5.38 (3.37–8.14)
All-cause death	2278	56	64.54	0.87 (0.66–1.13)
Control	1996	45	57.45	0.78 (0.57–1.05)
NDLVC	110	3	2.75	1.09 (0.23–3.19)
DCM	172	8	4.35	1.84 (0.79–3.62)
Heart failure	2278	48	63.79	0.75 (0.55–1)
Control	1996	29	56.97	0.51 (0.34–0.73)
NDLVC	110	4	2.73	1.46 (0.4–3.75)
DCM	172	15	4.09	3.67 (2.05–6.05)

*DCM* dilated cardiomyopathy, *NDLVC* non-dilated left ventricular cardiomyopathy

**Fig. 5:**
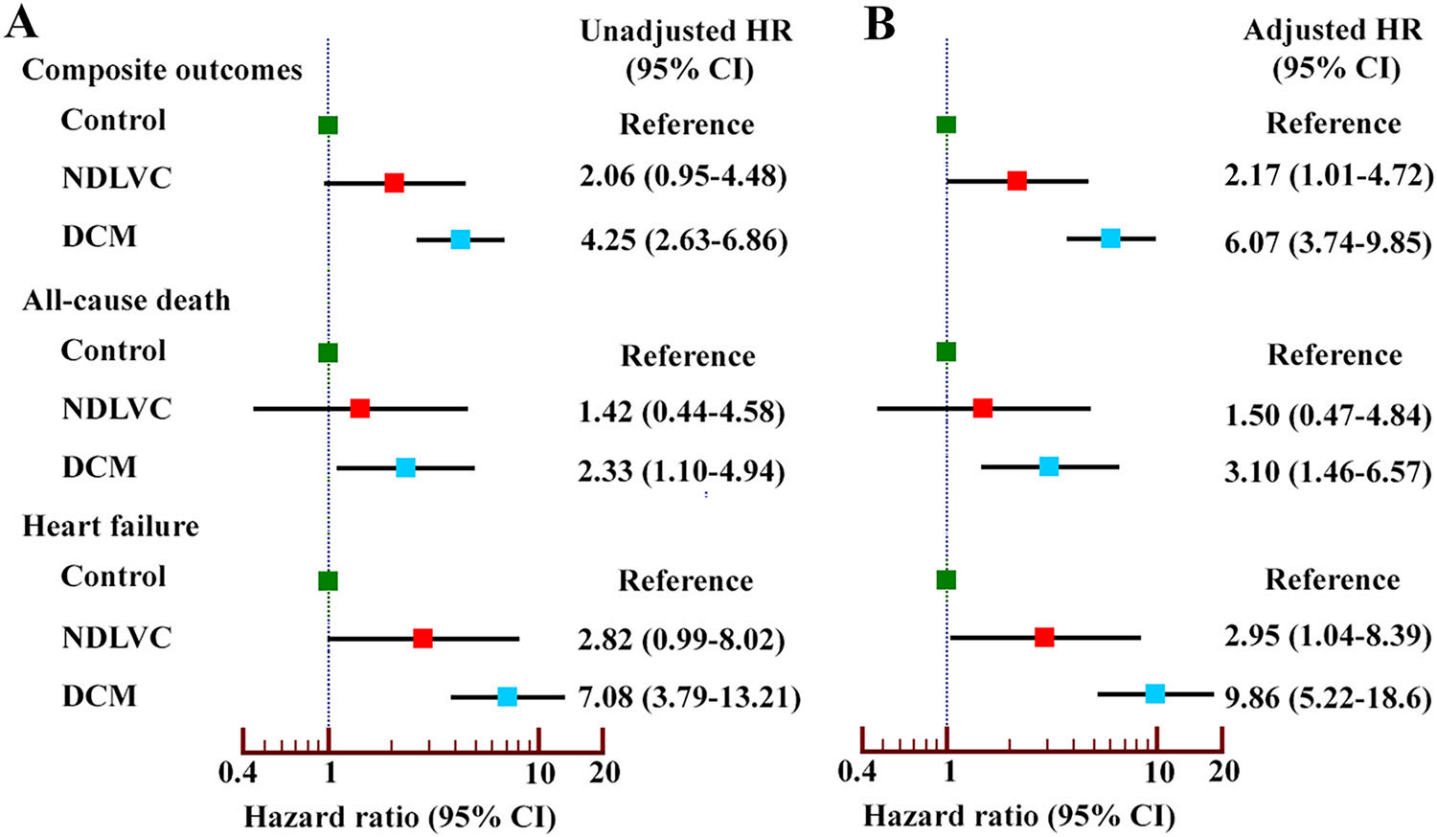
Forest plot displaying unadjusted (**A**) and adjusted (**B**) hazard ratios with 95% confidence intervals for non-dilated left ventricular cardiomyopathy and dilated cardiomyopathy, with the control group used as a reference

Figure 5 presents a forest plot showing unadjusted and adjusted hazard ratios and 95% CIs derived from univariate and multivariate Cox model analyses. These analyses compared the clinical outcomes of the DCM and NDLVC groups, using the control group as a reference. DCM had a greater risk of composite outcomes compared to NDLVC and controls, and NDLVC had a higher risk than controls (adjusted HR 6.07 (3.74–9.85) and 2.17 [1.01–4.72]). DCM patients had an increased risk of death and heart failure, whereas NDLVC had an increased risk of heart failure.

### CMR feature-tracking and ECV analysis

**Fig. 6:**
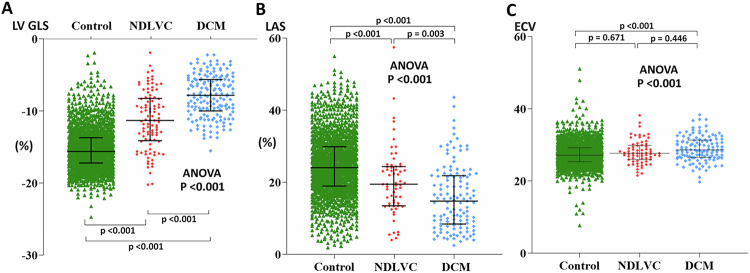
Scatter plots of left ventricular global longitudinal strain (**A**), left atrial strain (**B**), and extracellular volume fraction (ECV) (**C**) across the control, non-dilated left ventricular cardiomyopathy and dilated cardiomyopathy groups. The mean and standard deviation of the data are represented with the error bars. DCM, dilated cardiomyopathy; LAS, left atrial strain; LV-GLS, left ventricular global longitudinal strain; NDLVC, non-dilated left ventricular cardiomyopathy

According to the CMR feature-tracking analysis, LV-GLS among DCM patients was more impaired (−7.9 ± 2.9%) than that of the NDLVC patients (−11.3 ± 4.0%, *p* < 0.001) and the controls (−15.3 ± 2.8%, *p* < 0.001). The NDLVC group also had a greater LV-GLS numerically than the control group (*p* < 0.001; Fig. 6A). Regarding LAS, both DCM and NDLVC patients had lower LAS values than did the controls (15.8 ± 9.0% for DCM, 20.1 ± 9.5% for NDLVC, and 24.6 ± 8.4% for the controls, with *p* < 0.001 in both comparisons). Noteworthy, the DCM group exhibited a significantly lower LAS than the NDLVC group (*p* < 0.001; Fig. 6B).

DCM had a significantly greater ECV compared to controls (30.7 ± 4.8% vs 28.5 ± 5.3%, *p* < 0.001). There was no significant difference in ECV between patients with DCM and NDLVC (30.7 ± 4.8% vs 29.0 ± 4.7%, *p* = 0.004) and between NDLVC and controls (Fig. 6C).

### Sensitivity analysis

Sensitivity analysis was performed by the inclusion of controls without a history of heart failure, atrial fibrillation, or stroke. The unadjusted and adjusted hazard ratios and 95% CI for composite outcomes of DCM were 6.01 (3.58–10.10), *p* < 0.001, and 8.05 (4.77–13.57), *p* < 0.001, with the control group as the reference. The results were 2.91 (1.31–6.50), *p* = 0.009, and 3.05 (1.37–6.80), *p* = 0.006 for NDLVC compared to controls. The unadjusted and adjusted hazard ratios and 95% CI for heart failure outcome were 9.35 (4.71–18.56), *p* < 0.001, and 12.29 (6.15–24.57), *p* < 0.001, for DCM and 3.74 (1.26–11.04), *p* = 0.017, and 3.91 (1.32–11.57), *p* = 0.014, for NDLVC.

### Results of coronary angiogram (CAG) or coronary computerized tomography angiography (CCTA)

Coronary angiography (CAG) was performed in 129 (5.7%) of the study population (NDLVC, DCM, or control); 64 (49.6%) before CMR and 65 (50.4%) after CMR. The closest date of CAG was −37.3 ± 28.2 months for those who had CAG before CMR and 23.4 ± 19.0 months for those with CAG after CMR. Coronary computerized tomography angiography (CCTA) was performed in 78 (3.4%) of the study population; 42 (53.8%) before CMR and 36 (46.2%) after CMR. The closest date of CCTA was −21.1 ± 20.9 months for those who had CCTA before CMR and 33.3 ± 18.1 months for those with CCTA after CMR. A total of 195 (8.6%) patients had either CAG or CCTA. The results showed that 67 (34.4%) had a significant coronary artery stenosis defined as at least 50% stenosis in at least one of the major coronary arteries. For patients who had significant CAD, 6 (9.0%) had left main disease, 35 (52.2%), 21 (31.3%), and 10 (14.9%) had single vessel disease, double vessel disease, and triple vessel disease, respectively. Among patients who underwent CAG or CCTA, significant CAD was demonstrated in 9/20 (45.0%), 5/12 (41.7%), and 40/97 (41.2%) of patients with DCM, NDLVC, and control (*p* = 0.953), respectively.

## Discussion

The outcomes of this retrospective cohort study showed that among 4377 patients evaluated with CMR for stress perfusion or viability within the routine clinical practice of our center, the prevalence of NDLVC was 2.5%. Particularly, NDLVC represented a 39% among those who were traditionally classified as non-ischemic cardiomyopathy. NDLVC patients faced a greater risk of experiencing composite outcomes, primarily due to heart failure event, than did the controls, but with a lower risk than those with DCM. This increased risk in NDLVC patients persisted even after adjustment for potential confounders. Both the LV-GLS and LAS were also significantly impaired in patients with NDLVC, but to a lesser extent than in patients with DCM, indicating the substantial mechanical dysfunction within this disease spectrum.

The definition of NDLVC, which was in line with the ESC guidelines, included patients without LV dilation but with the presence of non-ischemic LGE, regardless of global or regional or systolic dysfunction or those with isolated LV hypokinesia without LGE [5]. Some NDLVC cases may represent an early stage of DCM, particularly in patients with mid-wall LGE, which may be evident prior to ventricular dilation [14]. Other patients with NDLVC may previously have myocarditis, as evidenced by patchy or subepicardial LGEs [14]. Approximately 40% of patients with DCM exhibit LGE [15, 16], which presents in various patterns, including mid-wall striae, and patchy subepicardial and even subendocardial LGE [15]. The presence of a mid-wall LGE in DCM patients is useful for diagnosis and has significant prognostic value [14, 15, 17]. In cases of myocarditis, the myocardial LGE pattern is typically subepicardial or patchy [18], occurring in approximately 70% of myocarditis cases [19]. Patients with myocarditis who exhibit LGE are also at an increased risk of adverse outcomes [20, 21].

In 2016, the ESC delineated ‘hypokinetic non-dilated cardiomyopathy’ (HNDC) as LV systolic dysfunction without LV dilation [6]. However, the 2023 ESC cardiomyopathy guidelines broadened the scope of HNDC to encompass patients with non-ischemic LGE even if they have normal LV function [5]. In a recent study of 785 patients characterized by LV systolic dysfunction (defined as LVEF < 45%), the prevalence of HNDC among patients with LV dysfunction was reported to be 20% [22]. In our study, NDLVC accounted for 39% of the combined DCM and NDLVC cohort, surpassing the previously reported 20% prevalence of HNDC in patients with LV dysfunction [22]. The description of HNDC was based on the presence of LV systolic dysfunction without dilatation [6]. This description did not include the LGE data from CMR, whereas NDLVC is defined as the presence of non-ischemic LGE (with or without global or regional wall motion abnormalities) or as isolated global LV hypokinesia without LGE [5]. Therefore, HNDC seems to be only a subset of NDLVC. This fact can explain why the proportion of NDLVC (39%) in our study is more common than HNDC (20%) among patients with LV systolic dysfunction.

The differences between the results of the previous study [22] and our study were (1) the previous study use echocardiography whereas our study used CMR which had more technologically advantages, (2) the previous study included only patients with LV systolic dysfunction and compared prevalence and prognosis of HNDC whereas our study had control group in addition to DCM and HNDC, (3) the previous study did not include heart failure endpoint but we did, (4) the previous study showed that mortality rate was not different between DCM and HNDC but our study also demonstrated a significantly higher mortality in DCM compared to controls, (5) we also demonstrated that the rate of heart failure are significantly higher in DCM compared to NDLVC.

Regarding clinical outcomes, this study revealed that patients with NDLVC faced an increased risk of composite outcomes, including death or heart failure events, compared with the controls, but exhibited a lower risk than did those with DCM. These results contrast with earlier findings on HNDC [22], where no significant differences were observed in the all-cause mortality, composite endpoints (mortality, heart transplantation, left ventricular assist device) of HNDC patients compared to those with DCM. The difference between our study and the study by Dziewięcka et al [22] was the use of different terms representing non-dilated spectrum of cardiomyopathy and the inclusion of different components of the composite outcomes. The previous study reported no difference in outcome between HNDC and DCM, whereas we reported an increased risk of both NDLVC and DCM compared to controls. DCM had a greater risk than NDLVC. The increased risk of DCM is related to both mortality and heart failure, while the elevated risk of NDLVC in our study was largely attributable to heart failure events. Patients with DCM had a significantly greater mortality risk than the controls (incidence rate 1.84 vs. 0.78 per 100 person-years, *p* = 0.021). However, the mortality rate for patients with NDLVC did not significantly differ from that of the controls (incidence rate 1.09 vs. 0.78 per 100 person-years, *p* = 0.275).

The results of the feature-tracking and ECV analysis using LV-GLS, LAS, and T1 mapping support the findings of the clinical outcome data. DCM group had significant abnormality in LV-GLS, LAS, and ECV compared to controls, and NDLVC is between DCM and controls, with significantly different from controls in LV-GLS and LAD and non-significant difference in ECV from control.

The wording ‘NDLVC’ represents a cardiomyopathy phenotype with could be the presence of non-CAD-related LV scarring (with or without global or regional wall motion abnormalities) or as isolated global LV hypokinesia without scarring without left ventricular dilatation [5]. The LGE patterns detected in patients with NDLVC have been reported in other types of myocardial diseases, such as chronic myocarditis [23], sarcoidosis [24], pulmonary hypertension [25], etc. Therefore, NDLVC is not the final diagnosis. Patients with NDLVC should be explored for the possibility of specific myocardial diseases or other conditions, such as pulmonary hypertension, that may be the underlying disease behind NDLVC. ACC/AHA does not have entity such as NDLVC. The scientific statement of the AHA, the definition of DCM required left ventricular dilatation and depressed myocardial systolic function [26]. They mentioned that patients with minimal left ventricular dilatation may have an early disease or recover after treatment. The prognosis also depends on the degree of left ventricular dilatation, which is consistent with the results of our study, that patients with NDLVC had a prognosis between patients with DCM and controls.

The population in this study included patients who were requested for CMR for the assessment of myocardial perfusion or viability. Therefore, in order to be suspected of coronary artery disease, most patients had some components of cardiovascular risk factors as shown in Table 1. Some risk factors may contribute to the increased risk of death or heart failure. The results of univariable analysis for the composite outcome of death or heart failure showed the hazard ratio and 95% CI of dyslipidemia, diabetes mellitus, hypertension and chronic kidney disease of 0.80 (0.54–1.19), 1.81 (1.23–2.69), 1.41 (0.89–2.25), and 2.52 (1.70–3.73). In the multivariable analysis, diabetes mellitus and chronic kidney disease remained in the final model with the hazard ratio and 95% CI of 1.88 (1.23–2.89) and 1.54 (1.01–2.34). They did not interfere with the significant predictive value of DCM and NDLVC for the outcomes.

The study’s limitations include its single-center nature, which potentially affects generalizability. Furthermore, the absence of genetic data made it impossible for us to conduct genomic analyses, which could have potentially refined predictions for clinical outcomes and cardiomyopathy classifications. Additionally, the absence of follow-up CMR data limited our understanding of the progression or potential transition of NDLVC to DCM in terms of myocardial structure or function. Moreover, despite patients, we excluded patients with a history of myocardial infarction, coronary revascularization, of evidence of CAD-pattern LGE or myocardial ischemia from CMR; 195 (8.6%) patients had either CAG or CCTA of whom 67 (2.9%) had a significant coronary artery stenosis defined as at least 50% stenosis in at least one of the major coronary arteries. This finding raises the possibility that some enrolled patients may have had undetected CAD even when negative for LGE and ischemia from CMR. Lastly, the prevalence of NDLVC and DCM in our cohort of patients applies to the group of patients with indications for CMR (mostly for exclusion of CAD). It is not applicable to the population of asymptomatic patients.

## Conclusions

NDLVC accounted for 2.5% of patients referred for CMR imaging for stress perfusion or myocardial viability assessments. Among those with LV systolic dysfunction without CAD or other specific types of cardiomyopathies, 39% had NDLVC. The risk of death or heart failure was greater in the NDLVC group than in the control group and was primarily driven by the incident of heart failure. Patients with NDLVC had an impaired LV-GLS and LAS compared to controls, but at a lesser extent than DCM. Patients with DCM had a significantly greater ECV compared to controls, but ECV of NDLVC and controls were not different.

## Abbreviations

CAD: Coronary artery disease
CMR: Cardiac magnetic resonance
DCM: Dilated cardiomyopathy
ECV: extracellular volume fraction
ESC: European Society of Cardiology
HNDC: Hypokinetic non-dilated cardiomyopathy
LAS: Left atrial strain
LGE: Late gadolinium enhancement
LVEF: Left ventricular ejection fraction
LV-GLS: Left ventricular global longitudinal strain
NDLVC: Non-dilated cardiomyopathy
